# Correlation between measures of insulin resistance in fasting and non-fasting blood

**DOI:** 10.1186/1758-5996-3-23

**Published:** 2011-09-07

**Authors:** Robert J Hancox, C Erik Landhuis

**Affiliations:** 1 Department of Preventive & Social Medicine, Dunedin School of Medicine, University of Otago, Dunedin, New Zealand; 2 Department of Social Sciences, Faculty of Applied Humanities, AUT University, Auckland, New Zealand

## Abstract

**Background:**

Epidemiological investigation of insulin resistance is difficult. Standard measures of insulin resistance require invasive investigations, which are impractical for large-scale studies. Surrogate measures using fasting blood samples have been developed, but even these are difficult to obtain in population-based studies. Measures of insulin resistance have not been validated in non-fasting blood samples. Our objective was to assess the correlations between fasting and non-fasting measures of insulin resistance/sensitivity.

**Methods:**

Fasting and non-fasting measurements of metabolic function were compared in 30 volunteers (15 male) aged 28 to 48 years. Participants provided a morning blood sample after an overnight fast and a second sample approximately 4 hours after lunch on the same day.

**Results:**

Non-fasting levels of the adipokines leptin, adiponectin, and leptin:adiponectin ratios were not significantly different and highly correlated with fasting values (r values 0.95, 0.96, and 0.95 respectively, P values < 0.001). There were moderate correlations between fasting and non-fasting estimates of insulin sensitivity using the McAuley (r = 0.60, P = 0.001) and QUICKI formulae (r = 0.39, P = 0.037). The HOMA-IR estimate of insulin resistance was also moderately correlated (r = 0.45, P = 0.016).

**Conclusions:**

Semi-fasting measures of leptin, adiponectin, and leptin:adiponectin ratios correlate closely with fasting values and are likely to be sufficient for population-based research. Other measures of insulin resistance or sensitivity in semi-fasted blood samples are moderately correlated with values obtained after an overnight fast. These estimates of insulin resistance/sensitivity may also be adequate for many epidemiological studies and would avoid the difficulties of obtaining fasting blood samples.

## Background

Assessment of insulin resistance presents a challenge for epidemiological research. The standard method to measure insulin resistance/sensitivity is the euglcaemic clamp technique, which requires infusions of insulin and glucose over about 2 hours with measurements of blood glucose every few minutes. The test is unsuitable for large-scale population-based studies because it is invasive, expensive, and time-consuming [1]. Hence a number of surrogate measures have been developed based on insulin, glucose and triglyceride levels in fasting blood [1,2]. Many of these surrogates correlate strongly with direct measurements of insulin resistance and are generally regarded as valid measures [3].

Even fasting blood tests can be problematic for population-based studies, however, because many participants may be unwilling to fast overnight before attending the study: for example, less than half of the participants followed the instruction to fast in the National Health and Nutrition Examination Survey [4]. Moreover, some participants may not admit to having eaten on the morning of the test increasing measurement variability due to the mix of fasting and non-fasting samples and introducing the potential for bias. Fasting also restricts scheduling of assessments because the blood tests are usually taken before breakfast and adds to the time and resources needed for the research because participants may need to be provided with food before undergoing further assessments.

Levels of adiponectin, and the ratio between leptin and adiponectin have also been proposed as surrogate markers of insulin resistance [5,6]. It has been suggested that the leptin:adiponectin ratio may be a stable indicator in non-fasted people since the fluctuations in their levels are modest [6-8]. Nevertheless, both are altered by prolonged fasting and studies reporting non-fasting measures of these adipokines have been criticised because of this [9].

It is unknown whether insulin resistance can be assessed using non-fasting blood. For most epidemiological purposes, the issue is not whether there are differences in the absolute values between fasting and non-fasting measures, but the extent to which non-fasting values reflect the underlying trait. We hypothesised that fasting and non-fasting metabolic indices would be highly correlated and, in preparation for an assessment of the 38 year-old participants of the Dunedin Multidisciplinary Health & Development Study,[10] sought to compare these in healthy adults.

## Methods

Volunteers aged 28-48 years were recruited. Pregnant women, people with diabetes, and those on medication likely to affect glucose or fat metabolism (e.g. oral corticosteroids) were excluded. Participants reported their height and weight.

Participants were instructed not to eat or drink anything other than water after midnight before the assessment day. They were asked to attend the laboratory for a blood test between 8 and 9 am. They were then provided with breakfast at a nearby restaurant. They were asked to eat lunch between 12 noon and 12.30pm on the same day and return for a second blood test between 4 and 4.30 that afternoon: approximately 4 hours after lunch. Drinks were permitted during the afternoon, but participants were asked to avoid drinks containing sugar and food. Participants were asked when they had last eaten when they attended for each blood test. No restrictions were placed on the participants' physical activity during the assessment day. To obtain an indication of the short-term repeatability of the tests, 10 participants also provided a second fasting morning blood sample, 1 week to 2 months later.

Blood samples were obtained while seated and were analysed for glucose and triglycerides on a Roche Modular analyser (Roche Diagnostics, Mannheim, Germany) using enzymatic assays. Plasma leptin was measured by radioimmunoassay (HL81K, Linco Research, St. Charles MO, USA: coefficient of variation 7%). Adiponectin was measured by enzyme-linked immunosorbent assay (ARCP30, Millipore, St. Charles MO, USA: coefficient of variation less than 8.4%) Samples for insulin were placed on ice immediately after being drawn. Plasma free insulin was measured on a Roche Elecsys 2010 analyser (Roche Diagnostics: coefficient of variation 3.8%) after polyethylyene glycol precipitation of interfering antibodies.

Estimates of insulin sensitivity were calculated using the McAuley formula (exp(2.63-0.28 × ln(insulin) - 0.31 × ln(triglyceride)),[2]. and Quantitative Insulin Sensitivity Check Index (QUICKI: 1/(ln(insulin)+ln(glucose)) [11]. The Homeostasis Model Assessment of Insulin Resistance (HOMA-IR) was determined from glucose and insulin values using the online calculator [12,13]. Leptin, adiponectin, insulin, HOMA-IR, and leptin:adiponectin ratio values were log-transformed to approximate normal distributions. The primary outcome of this analysis was the sex-adjusted partial correlation coefficient between the fasting and non-fasting values. Fasting and non-fasting values were also compared using paired t-tests.

A sample size of 30 was calculated to provide sufficient power to detect significant correlations between samples of r = 0.3 or greater. Correlations smaller than this would be unlikely to be of interest to epidemiological researchers wishing to find a surrogate measure for fasting values. The study was approved by the University ethics committee. All participants provided written informed consent.

## Results

15 men and 15 women aged 28 to 48 years (mean 39.6) were recruited. The mean self-reported Body Mass Index was 25.3 kg/m^2 ^(range 20 to 32). Fasting times for the morning blood test were between 9 hours 25 minutes and 14 hours 43 minutes (mean 12:24). Time since last food before the afternoon blood test varied from 2 hours 30 minutes to 5 hours 55 minutes (mean 3:55). 5 participants had eaten within 3 hours 30 minutes of this test.

Triglycerides were higher in non-fasting samples and there was a trend to higher insulin levels. Glucose, leptin, and adiponectin were not significantly different. Mean levels and sex-adjusted correlation coefficients between fasting and non-fasting values are shown in table 1.

**Table 1:** Mean Fasting and Non-Fasting Values and Correlation Coefficients

	Comparison of means^a^					Sex-adjusted partial correlation	
	Fasting		Non-Fasting				
Variable	Mean	95% CI	Mean	95% CI	P	r	P
Glucose mmol/L	5.06	4.88, 5.24	4.98	4.80, 5.16	0.44	0.40	0.033
Insulin^b ^pmol/L	31.8	25.4, 39.9	40.8	29.1, 57.3	0.11	0.44	0.016
Triglyceride mmol/L	0.89	0.74, 1.04	1.52	1.22, 1.81	< 0.001	0.65	< 0.001
Leptin^b ^μg/L	5.81	4.09, 8.24	6.00	4.33, 8.3	0.37	0.95	< 0.001
Adiponectin^b ^μg/L	7.25	5.88, 8.93	6.45	4.51, 9.23	0.17	0.96	< 0.001
McAuley	10.0	9.1, 10.9	8.3	7.1, 9.6	0.002	0.60	0.001
QUICKI	0.17	0.16, 0.17	0.16	0.15, 0.17	0.41	0.39	0.037
HOMA-IR^b^	0.60	0.48, 0.75	0.76	0.54, 1.07	0.12	0.44	0.016
Leptin:adiponectin ratio^b^	0.83	0.60, 1.15	0.83	0.61, 1.13	0.89	0.95	< 0.001

^a ^P values from two-sided paired t-tests. ^b ^Values log-transformed before analyses: geometric means are presented.

Calculated insulin sensitivity using the McAuley formula was lower for the afternoon non-fasting blood sample, but highly correlated with the fasting measure (Table 1). Fasting and non-fasting values for QUICKI and HOMA-IR were moderately correlated. Leptin:adiponectin ratios were similar in fasting and non-fasting samples and were very highly correlated.

## Discussion

This study demonstrates strong and significant correlations between fasting and non-fasting blood tests for several metabolic indicators. These findings suggest that it may be acceptable to use non-fasting bloods to assess insulin resistance in epidemiological studies. This would avoid a number of difficulties inherent in asking participants to fast overnight and may remove one of the barriers to participation in these studies.

The adipokine measurements were the most stable measures between fasting and non-fasting samples. Non-fasting levels of leptin, adiponectin, and the leptin:adiponectin ratio were not significantly different and were very strongly correlated with the fasting values. Hence, we can confirm that these do not require fasting samples. Although adipokines levels have previously been shown to be influenced by fasting, this appears to require prolonged food deprivation [14].

As expected, triglycerides were higher in non-fasting samples. Despite this, there was a strong correlation between the fasting and non-fasting values. More importantly, estimates of insulin sensitivity derived from non-fasting insulin and triglyceride levels using the McAuley formula were moderately strongly correlated with fasting estimates (r = 0.6). Correlations of this magnitude may be adequate for many epidemiological purposes.

We did not compare non-fasting measures of insulin resistance with the so-called "gold-standard" technique: the euglycaemic clamp [1]. A fasting measure of insulin resistance using the McAuley formulae correlates moderately well with this measure and it is plausible that a non-fasting version of this formula would not correlate as well [2,3]. However, all methods of assessing insulin resistance have limitations and problems with repeatability [1]. In fact the correlation between same-day fasting and non-fasting samples for the McAuley formula was similar to the correlation between two fasting samples measured 1 week to 8 weeks apart in 10 participants who provided a second fasting sample, suggesting that non-fasting estimates may be no worse than the short-term repeatability of the fasting estimate. Other estimates of insulin sensitivity or resistance (QUICKI and HOMA-IR, both of which are based on insulin and glucose measurements) were also moderately correlated between fasting and non-fasting samples.

It should be noted that the finding that fasting and non-fasting measures were correlated does not mean that the measures are equivalent: there were differences in the mean values of some of the measurements despite the correlations between them. We also do not know if a change in the value of one of these measurements over time will necessarily be accompanied by an equivalent change in the other and it remains possible that they reflect different aspects of the underlying metabolic trait. However, these considerations apply to all surrogate markers of insulin resistance whether they are fasting or not [15].

A number of methodological issues deserve comment. This study was part of the preparation for an assessment of 38 year-olds [10]. Hence the age range was restricted to age 38 +/-10 years to avoid false inflation of the correlations between fasting and non-fasting samples due to age-related differences (adjusting the correlation coefficients for age made no material difference to the findings). We purposefully made no attempt to influence the content or quantity of the mid-day meal prior to the non-fasting blood test. It is difficult to standardise meals in population-based studies because of differences in food preferences and appetite. Therefore we decided to let the participants chose their own lunch to make a realistic assessment of the variability between fasting and non-fasting values. We also permitted non-sugary drinks (including tea and coffee with milk), but not food, in the four hours before the afternoon non-fasting test. We believe that this is what might reasonably be achieved in a population-based study. Our findings may have been different if the participants had been permitted to eat during this time. In fact, despite being asked to avoid food for approximately 4 hours before the afternoon blood test, 5 participants had consumed food less than 3 hours 30 minutes before. Omitting these participants from the analysis improved the correlations between fasting and non-fasting values for triglycerides (r = 0.80), insulin (r = 0.45), McAuley (r = 0.76), HOMA-IR (r = 0.62), and QUICKI (r = 0.56). This observation suggests that the 4-hour fast is important, although more data are needed to confirm this. Achieving a 4-hour semi-fasting state is likely to be much easier for researchers and more acceptable to participants than an 8 - 10 hour overnight fast. This does not require participants to miss breakfast, and most people are accustomed to waiting 4 hours between meals. In addition, many research protocols take more than 4 hours to complete, permitting the researchers to monitor whether the participants have observed the request to avoid eating during this time.

## Conclusions

There are strong correlations between estimates of insulin resistance and sensitivity in fasting and non-fasting blood samples. Leptin, adiponectin and the leptin:adiponectin ratio did not differ and were strongly correlated between fasting and non fasting samples. The findings suggest that fasting samples are not required for measurement of adipokines and that standardised semi-fasting blood tests to estimate insulin resistance may be adequate for many epidemiological purposes.

## Abbreviations

**HOMA-IR**: Homeostasis Model Assessment of Insulin Resistance; **QUICKI**: Quantitative Insulin Sensitivity Check Index.

## Competing interests

Neither author has a conflict of interest with the contents of this manuscript.

## Authors' contributions

RJH conceived of the study, collected and analysed the data, and drafted the manuscript. CEL participated in the study design, collected data, and critically reviewed the manuscript. Both authors read and approved the final manuscript.
